# Enhanced capacity of thiol-functionalized sugarcane bagasse and rice husk biochars for arsenite sorption in aqueous solutions

**DOI:** 10.1007/s11356-024-34661-4

**Published:** 2024-08-15

**Authors:** Israr Masood ul Hasan, Nabeel Khan Niazi, Irshad Bibi, Fazila Younas, Fahad Al-Misned, Muhammad Bilal Shakoor, Fawad Ali, Shazia Ilyas, Muhammad Mahroz Hussain, Jinli Qiao, Andreas Lüttge

**Affiliations:** 1 Institute of Soil and Environmental Sciences, University of Agriculture Faisalabad, Faisalabad, 38040 Pakistan; 2 State Key Laboratory for Modification of Chemical Fibers and Polymer Materials, College of Environmental Science and Engineering, Donghua University, 2999 Ren’min North Road, Shanghai, 201620 China; 3 Key Laboratory of Comprehensive and Highly Utilization of Salt and Lake Resources, Qinghai Institute of Salt and Lakes, Chinese Academy of Sciences, Xining, 810008 China; 4 Department of Geosciences and MARUM—Center for Marine Environmental Sciences, University of Bremen, 28359 Bremen, Germany; 5 School of Geography, Earth and Atmospheric Sciences, University of Melbourne, Melbourne, VIC 3053 Australia; 6 School of Environmental Science and Engineering, Shandong University, Qingdao, 266237 China; 7 Department of Zoology, College of Science, King Saud University, 11451 Riyadh, Saudi Arabia; 8 College of Earth and Environmental Sciences, University of the Punjab, Lahore, 54000 Pakistan; 9 Centre for Planetary Health and Food Security, Griffith University, Nathan Campus, Nathan 4111, Brisbane, Queensland Australia; 10 Queensland Department of Agriculture and Fisheries (QDAF), Mareeba 4880, Brisbane City, Queensland Australia; 11 Department of Environmental Sciences, Forman Christian College (A Chartered University), Lahore, 54600 Pakistan; 12 Shanghai Institute of Pollution Control and Ecological Security, Shanghai, 200092 China; 13 College of Environmental Science and Engineering, Yangzhou University, Yangzhou, 225127 China

**Keywords:** Biowastes, Contamination, Drinking water treatment, Waste management

## Abstract

The utilization of biowastes for producing biochar to remove potentially toxic elements from water represents an important pathway for aquatic ecosystem decontamination. Here we explored the significance of thiol-functionalization on sugarcane bagasse biochar (Th/SCB–BC) and rice husk biochar (Th/RH–BC) to enhance arsenite (As(III)) removal capacity from water and compared their efficiency with both pristine biochars (SCB–BC and RH–BC). The maximum As(III) sorption was found on Th/SCB–BC and Th/RH–BC (2.88 and 2.51 mg g^−1^, respectively) compared to the SCB–BC and RH–BC (1.51 and 1.40 mg g^−1^). Relatively, a greater percentage of As(III) removal was obtained with Th/SCB–BC and Th/RH–BC (92% and 83%, respectively) at a pH 7 compared to pristine SCB–BC and RH–BC (65% and 55%) at 6 mg L^−1^ initial As(III) concentration, 2 h contact time and 1 g L^−1^ sorbent dose. Langmuir (*R*^2^ = 0.99) isotherm and pseudo-second-order kinetic (*R*^2^ = 0.99) models provided the best fits to As(III) sorption data. Desorption experiments indicated that the regeneration ability of biochars decreased and it was in the order of Th/SCB–BC (88%) > Th/RH–BC (82%) > SCB–BC (77%) > RH–BC (69%) up to three sorption–desorption cycles. Fourier-transform infrared spectroscopy and X-ray photoelectron spectroscopy results demonstrated that the thiol (-S–H) functional groups were successfully grafted on the surface of two biochars and as such contributed to enhance As(III) removal from water. Spectroscopic data indicated that the surface functional moieties, such as -S–H, − OH, − COOH, and C = O were involved to increase As(III) sorption on thiol-functionalized biochars. This study highlights that thiol-grafting on both biochars, notably on SCB–BC, enhanced their ability to remove As(III) from water, which can be used as an effective technique for the treatment of As from drinking water.

**Graphical Abstract:**

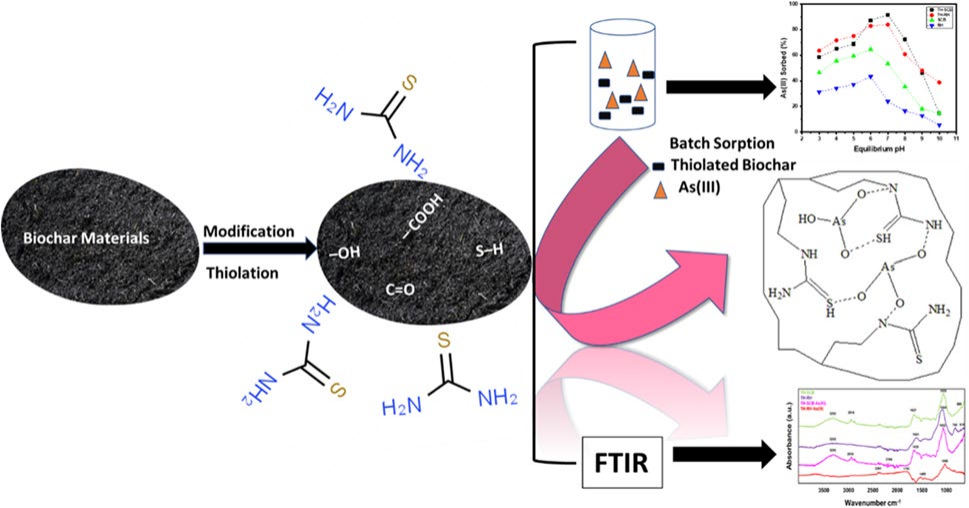

**Supplementary Information:**

The online version contains supplementary material available at 10.1007/s11356-024-34661-4.

## Introduction

Groundwater arsenic (As) contamination has become an alarming issue worldwide because As is classified as a Class 1 human carcinogen due to its toxic properties (Mukherjee et al. 2020; Song et al. 2023). Arsenic is released in groundwater, surface water, soil and sediments both geogenically (such as weathering of minerals, geothermal waters) and anthropogenically, including pesticides and fertilizer application, smelting and mining activities, coal combustion, and chromium-copper-arsenate (CCA) treatment of wood (Niazi et al. 2023). Global As contamination, mainly from drinking As-contaminated groundwater, have led to the poisoning of over 200 million people, especially in South and Southeast Asia (e.g., Pakistan, Bangladesh, China, and India) and South America (Amen et al. 2020). Arsenic occurs in organic and inorganic forms, of which the inorganic form of As is 60 times more toxic than organic As species. Arsenic exists in two inorganic species, arsenite (As(III)) and arsenate (As(V)), with As(III) predominating under reducing aquatic environments and more toxic than As(V) (Hussain et al. 2021; Shakoor et al. 2019). Considering the toxic nature of As, the World Health Organization (WHO) recommended a safe threshold value of As in drinking water at 10 µg L^−1^ (WHO 2011). Various conventional techniques have been employed to remediate As-contaminated water, such as ion exchange, electrochemical, precipitation, membrane separation, and solvent extraction (Back et al. 2018, Amen et al. 2020). Most of these conventional techniques incur substantial capital and operational costs and secondary sludge pollution generation, thus making them challenging for successful execution in developing countries. To ensure universal access of clean water in alignment with the United Nation’s Sustainable Development Goal 6 (United Nations, 2015), there is a dire need to investigate new water treatment technologies for As. In recent research, numerous adsorbents, such as metal–organic frameworks (Noraee et al. 2019; Fortoutan et al. 2022), functionalized polymers (Anito et al. 2020) and graphene-based materials (Ahmad et al. 2020) have been used for the treatment of toxic elements and organic pollutants containing water. Various nanomaterials (Anjum et al. 2019; Wadhawan et al. 2020), biosorbents (Qin et al. 2020; Rambabu et al. 2023), and biochars (Amen et al. 2020; Ugwu and Agunwamba 2020) have also been used to remove As from contaminated water.

Chemical modification may enhance the adsorption capacity of biochar by changing surface properties, such as specific area and surface functional groups (e.g., phenolic, carboxyl) (Chai et al. 2010; Jiang et al. 2016; Zhang et al. 2012). Moreover, the thiol (S − H) functional group may enhance the bonding ability for anionic metal(loid) ions, such as As, more than the amino groups, oxygenic functional groups, and halogen atoms (Kazemi et al. 2016). Grafting of the − S−H group on the biochar may increase its sorption capacity for As(III) oxyanions because of Lewis acid–base interaction. Therefore, the integration of the − S − H surface functional group on biochar could be appraised as an excellent approach for As(III) removal from water.

Some previous studies support the − S−H applications on different sorbents (e.g., iron-based nanoparticles) for removal of metal ions (Pb, Hg, Ag, Cd) from water (Gan et al. 2016; Li et al. 2011; Shin and Jang 2007; Yantasee et al. 2007; Xia et al. 2019). In contrast, a few studies have been directed to examine the role of − S−H for As removal in aquatic systems. For instance, Yang et al. (2015) fabricated thiol-modified chitin nanofiber adsorbent for the removal of As from aqueous solutions. The as-prepared thiol-modified chitin nanofiber showed higher adsorption for As at neutral pH than all the existing chitin/chitosan-based adsorbents. Singh et al. (2018) reported that thiol-iron (TH–Fe) biofunctionalized composite removed 99% As from solutions containing As(V) and As(III). However, the intriguing significance of thiol functionalization on biochar for As(III) sorption, such as sugarcane bagasse and rice husk biochars, has not been explored previously. In addition, selective and enhanced sorption of As(III) species by thiolated biochars represents a critical advancement, while previously not investigated, for filtration of As from contaminated water. In this study, we explored and compared the potential of two newly developed thiol-functionalized sugarcane bagasse and rice husk biochars (Th/SCB–BC and Th/RH–BC) for As(III) removal from water, as well as compared the performance with their pristine biochars (SCB–BC and RH–BC).

## Materials and methods

### Materials

For the preparation of stock and sub-stock solutions, sodium arsenite (NaAs^III^O_2_; 99%) salt and deionized water were used, and 100 mg L^−1^ stock solution of As(III) was prepared to prepare working As(III) solutions for batch experiments. A 0.1 M hydrochloric acid (HCl; 35%) or sodium hydroxide (NaOH; 95%) was used to adjust the pH of the solution as required (Shakoor et al. 2019). All the chemicals used were of analytical grade (Sigma-Aldrich) for batch sorption experiments. Other chemical reagents, such as sodium chloride (NaCl; 99%), nitric acid (HNO_3_; 69%), ethanol (C_2_H_6_O; 99%) (BDH), and thiourea (CH_2_N_2_S; 99%) (Merck), were used as received without further purification. The plasticware and glassware were used after washing with tap water followed by soaking in 1% HNO_3_ and rinsing two times with deionized water.

### Collection of biowastes and preparation of biochars

Sugarcane bagasse (SCB) and rice husk (RH) wastes were collected from the local market of Faisalabad, Punjab (Pakistan) (31.4504° N; 73.1350° E). In recent years, agriculture biowastes, especially SCB and RH have emerged as cost-effective and alternative biomaterial for adsorbents (e.g., biochar, biosorbents, activated carbon) because of their availability in large quantities (Sen et al. 2023). Additionally, these biowastes are naturally and cheaply available in large quantities, i.e., the sugarcane industry produces large amounts of SCB, and RH is a huge waste from the rice processing industry, particularly in developing nations like Pakistan (Shaheen et al. 2022). These biowastes require minimal initial processing time and have negligible commercial value, and in most cases, these are burnt in the industry to produce energy, thus causing air pollution. Rice husk and SCB were washed with deionized water to remove dirt, dried in an oven at 65°C for 12 h, and ground (< 1 mm) for homogeneity. Rice husk and SCB were used to produce sugarcane bagasse biochar (SCB–BC) and rice husk biochar (RH–BC) by pyrolysis at 500 °C under a oxygen-limited environment with a residence time of 2 h in a closed furnace (Niazi et al. 2018a; Yuan and Xu 2012).

#### Preparation of thiol-functionalized biochars

In the preparation of thiol-functionalized SCB biochar (Th/SCB–BC) and thiol-functionalized RH biochar (Th/RH–BC), the pristine SCB and RH biochars were washed with HNO_3_ and heated in an oven at 65°C for 8 h. The SCB–BC and RH–BC were used for the thiol functionalization process. Based on the optimal ratio of biochar to thiourea (CH_2_N_2_S) (Singh et al. 2016) and using baseline information from our preliminary data (data not shown), 7:3 was used as the optimum biochar to CH_2_N_2_S ratio for high As(III) removal. The 50 g SCB–BC or RH–BC were dispersed in 70% C_2_H_6_O solution in a 1:10 ratio of sorbent to C_2_H_6_O followed by the addition of 0.5 M CH_2_N_2_S in a ratio of 7:3 (biochar to CH_2_N_2_S solution). The thiol-functionalized biochar was cooled and washed twice with C_2_H_6_O to remove excessive CH_2_N_2_S and dried again for 1 day at 60°C as reported by Singh et al. (2018).

### Characterization of sorbents

The surface morphology of sorbents was analyzed using scanning electron microscopy (SEM, Hitachi S4800, Japan). X-ray photoelectron spectroscopy (XPS, Thermo Escalab 250Xi USA) was used to determine the oxidation state and binding of As with surface functional groups.

Surface functional groups were analyzed using the Fourier-transform infrared (FTIR) spectroscopy (ATR-FTIR; Alpha-II, Bruker, Germany) with scans at 4000–600 cm^−1^ wavenumber range with ten successive scans at a resolution of 4 cm^−1^. Absorbance spectra were normalized and interpreted using the OPUS software (version 8.5.29).

X-ray diffraction (XRD) (Bruker D8 ADVANCE diffractometer, Germany) was used to determine crystalline mineral phases, if any, in biochars.

### Batch sorption experiments

A series of batch sorption experiments were carried out to determine the effect of SCB–BC, RH–BC, Th/SCB–BC, and Th/RH–BC on As(III) sorption under varying pH (3–10), contact time (0–24 h), sorbent dose (1–16 g L^−1^), and at initial As(III) concentration ranging from 0.05 to 10 mg L^−1^. In 50 mL plastic vials, sorption experiments were performed using 0.01 M NaCl solution as a background electrolyte. All the batch sorption studies were carried out at 20 ± 2 °C and a contact time of 2 h. In the case of the kinetic study, contact time varied from 0 to 24 h. The pH of solutions was maintained by using 0.1 M NaOH or HCl solution as required.

The effect of sorbent dose (1–16 g L^−1^) and pH (3–10) was examined on As(III) sorption by SCB–BC, RH–BC, Th/SCB–BC, and Th/RH–BC at a constant initial As(III) concentration (6 mg L^−1^). Different time intervals varying between 0.016 and 24 h were used for kinetic experiments at an optimum pH. Sorption isotherm experiments were executed at varying initial As(III) concentration (0.05 to 10 mg L^−1^) (Niazi et al. 2018b) at a sorbent dose of 1 g L^−1^ for all the biochars.

An end-to-end shaker was used to agitate the suspensions for 2 h gently at 35 rpm. After shaking, the suspensions were centrifuged at 4000 rpm for 10 min in a centrifuge machine (ROTOFIX 32 A, Germany). A 0.45-µm syringe filter was used to separate the liquid from the solid residue. The equilibrium pH was measured and filtered samples were kept at 4°C prior to As analysis using a hydride generation-atomic absorption spectrometer (HG-AAS; Agilent AA 240 with VGA–77; Australia) (Shakoor et al. 2018).

Removal percentage (%) of As(III) was calculated using Eq. (1) (Shakoor et al. 2018) as follows:

1 $$\%\;As\;removal= \frac{{C}_{O}-{C}_{e}}{{C}_{O}} \times 100$$

*C*_o_ represents the initial As(III) concentration (mg L^−1^) and *C*_e_ is the final As(III) concentration at equilibrium (mg L^−1^).

The sorption capacity (*q*_e_; mg g^−1^) was calculated at equilibrium using the following equation (Eq. (2)) (Shakoor et al. 2018):

2 $${q}_{e}=\frac{\left({C}_{O}- {C}_{e}\right) V}{m}$$

where *V* is the volume of solution (L), *m* is the oven-dried weight of sorbent (g), and *C*_o_ and *C*_e_ have been explained above.

### Desorption experiments

Desorption studies are important for evaluating the regeneration ability of four biochars investigated here. Arsenite desorption was carried out up to three sorption–desorption cycles using 0.2 M NaOH as an eluent solution. After desorption experiments, the sorbents were filtered and 25 mL eluent solution was added from 0.2 M NaOH followed by shaking for 40 min. The sorbent was separated from the mixture and stored at 4°C in a refrigerator for As analysis. Arsenic concentration was determined in filtered water samples after each sorption–desorption cycle using a HG–AAS as mentioned above.

### Sorption isotherm and kinetic modeling

Four sorption isotherm models, Langmuir, Freundlich, Temkin, and Dubinin–Radushkevich were used to examine As(III) sorption on biochars as described earlier (details in Supplementary Information) (Ahmad et al. 2014; Niazi et al. 2018a; Shakoor et al. 2018). Kinetic models (pseudo-first-order and pseudo-second-order) were used to determine the rate of sorption (Niazi et al. 2018a; Prasad et al. 2013). Equations (3) and (4) used for kinetic models are given below for pseudo-first-order and pseudo-second-order models, respectively:

3 $$log\left({q}_{e}-{q}_{t}\right)=log\;{q}_{e}-{k}_{1}.\frac{ t }{2.303}$$

4 $$\frac{t}{{q}_{t}}=\frac{1}{{k}_{2}{q}_{e}^{2}}+\frac{t}{{q}_{e}}$$

where *q*_e_ and *q*_t_ stand for sorbed As at equilibrium at any time (*t*); *k*_1_ and *k*_2_ are rate constants for pseudo-first-order and pseudo-second-order models, respectively.

### Arsenic analysis quality assurance and quality control

To maintain quality assurance of the analysis, reagent blanks (*n* = 3) were used and after every ten samples a known sample a reference standard with known As concentration was also run for quality control and analytical precision. For HG–AAS analysis, the residual standard deviation (RSD) was below 2.8%.

## Results and discussion

### Influence of pH

**Fig. 1:**
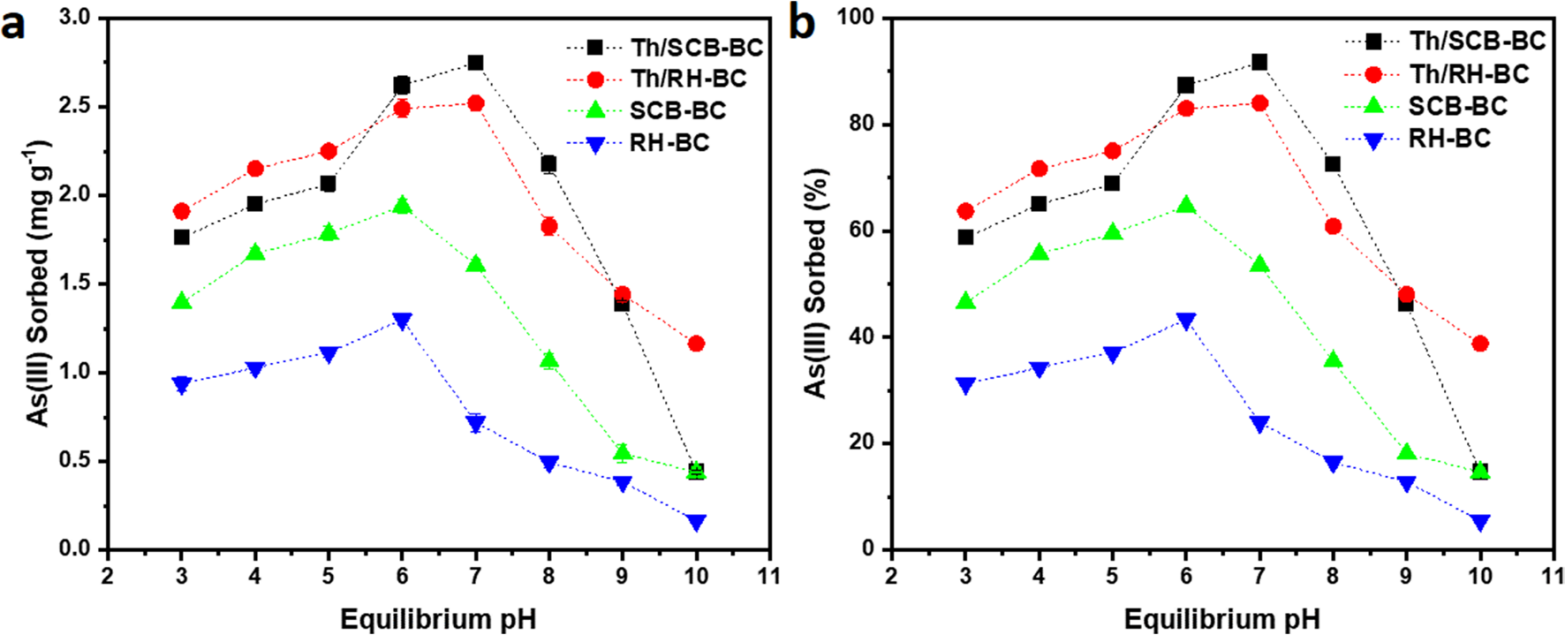
Effect of pH on (**a** and **b**) arsenite (As(III)) sorption by thiolated sugarcane bagasse biochar (Th/SCB–BC), Th/rice husk biochar (Th/RH–BC), sugarcane bagasse biochar (SCB–BC), and rice husk biochar (RH–BC) at an initial As(III) concentration of 6 mg L^−1^, sorbent dose of 1 g L^−1^, and at 20 °C

The effect of pH on As sorption and mobilization is an important parameter due to its effect on As species present in water (Amen et al. 2020). Results indicated that the removal efficiency of pristine and thiol-functionalized biochars for As(III) was pH dependent, with a gradual decrease in As sorption with pH increase from 3 to 7 and then a decrease with increasing pH from 8 to 10 (Fig. 1). Th/SCB–BC showed the highest As(III) sorption (2.75 mg g^−1^) with 92% removal from water followed by Th/RH–BC (2.43 mg g^−1^, 83%) at pH 7 (Fig. 1). In the case of pristine biochars, SCB–BC showed slightly higher removal efficiency (1.94 mg g^−1^, 65%) compared to RH–BC (1.65 mg g^−1^, 55%) for As(III) at pH 6. These results are in agreement with previous studies where thiol-functionalized activated carbon /alumina (80%) and TH–Fe (99.5%) showed maximum As(III) adsorption at neutral pH (Hao et al. 2009; Singh et al. 2018).

Higher sorption of As(III) on thiolated biochars (Th/SCB–BC, Th/RH–BC) than unthiolated biochars (SCB–BC and RH–BC) could be attributed to strong binding between As(III) and − S–H groups (Sing et al. 2018; Hao et al. 2009) Arsenite can form stable complexes with − S − H groups in aqueous solutions (Hao et al. 2009; Rey et al. 2004) under varying environmental conditions, such as solution pH. The maximum As(III) sorption near neutral pH could be due to less or no competition of OH anions with H_3_AsO_3_ species at pH 7 (Bibi et al. 2017).

### Effect of sorbent dose

**Fig. 2:**
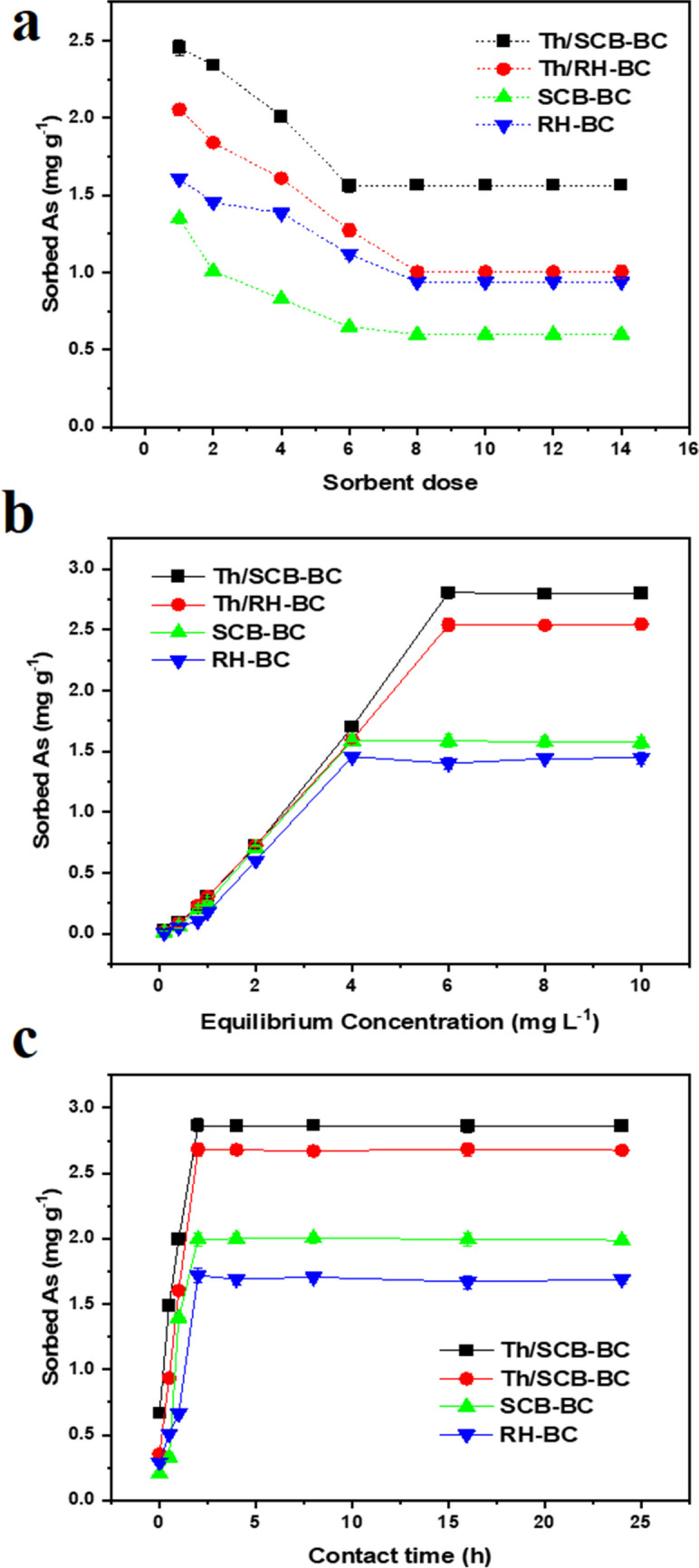
Effect of various factors on arsenite (As(III)) sorption by sugarcane bagasse biochar (SCB–BC), rice husk biochar (RH–BC), thiolated SCB–BC (Th/SCB–BC), and thiolated RH–BC (Th/RH–BC); **a** sorbent dose at initial As(III) concentration of 6 mg L^−1^, pH 7, and at 20 °C; **b** Initial As(III) concentration (6 mg L^−1^) at sorbent dose of 1 g L^−1^, pH 7, 2 h, and 20 °C; and (**c**) contact time at initial As(III) concentrations of 6 mg L^−1^, sorbent dose of 1 g L.^−1^, pH 7, and temperature at 20 °C. Mean values are shown as mean ± standard error (*n* = 3)

Maximum sorption occurred at sorbent dose of 1 g L^−1^ on Th/SCB–BC (2.45 mg g^−1^) and Th/RH–BC (2.05 mg g^−1^), which was 82% and 70%, respectively, compared to unthiolated (SCB–BC = 45% (1.35 mg g^−1^); RH–BC = 53% (1.60 mg g^−1^)) (Fig. 2a). At a high sorbent dose, low As(III) sorption was noted which could be due to the presence of unsaturated binding sites (Masood ul Hasan et al. 2022; Sattar et al. 2019). A compression of As(III) sorption of Th/SCB–BC, Th/RH–BC, SCB–BC, and RH–BC found in this study with some previous studies is shown in Table S1 Supplementary Information.

### Initial As(III) concentration

The effect of the initial As(III) concentration (0.05–10 mg L^−1^) was studied at an optimum pH (selected from the pH experiment) (Fig. 2b). Th/SCB–BC (2.8 mg g^−1^) and Th/RH–BC (2.5 mg g^−1^) showed the highest sorption capacity, with 93.5% and 84.7% As removal, respectively, at an initial concentration of 6 mg L^−1^. SCB–BC (1.58 mg g^−1^) and RH–BC (1.45 mg g^−1^) showed the highest sorption at an initial concentration of 4 mg L^−1^, with As removal of 79.2% and 72.7%, respectively. As the initial As(III) concentration increased from 0.05 to 6 mg L^−1^, the sorption of As enhanced. However, further increase in As concentration (from 6 to 10 mg L^−1^) indicated no influence on As(III) sorption for all the biochars. High As(III) sorption at low concentration could possibly be due to the large number of available active sites on sorbent with surface functional groups in the presence of As anions (Masood ul Hasan et al. 2022; Shakoor et al. 2018).

### Contact time

Results showed that the maximum As(III) sorption (2.86, 2.68, 1.99, and 1.72 mg g^−1^) was observed at 2 h contact time by Th/SCB–BC, Th/RH–BC, SCB–BC, and RH–BC, respectively (Fig. 2c). In the beginning (0–30 min) the sorption occurred fast and As(III) removal increased rapidly. After reaching an equilibrium time (2 h), As(III) sorption remained constant up to 4 h and then slightly decreased until 24 h time (Shaikh et al. 2020; Prasad et al. 2022).

### Sorption kinetics modeling

Table 1 shows the kinetic model parameters for As(III) sorption on Th/SCB–BC, Th/RH–BC, SCB–BC, and RH–RH surfaces (see Figs. S1 and S2, Supplementary Information). Data showed that the pseudo-second-order model (*R*^2^ = 0.99) provided the best fit for As(III) kinetics data for all the unthiolated and thiol-grafted biochars. The pseudo-second-order model *q*_e_ (cal) value not only provided a better fit to As(III) sorption kinetics but was also close to the experimental value compared to the pseudo-first-order model. Overall, kinetics modeling described that As(III) sorption on Th/SCB–BC, Th/RH–BC, SCB–BC, and RH–BC followed preferably pseudo-second-order and *q*_e_ values indicated that sorption was controlled by a rate-limiting step and involves interaction between surface functional groups and As oxyanions (Sattar et al. 2019).

**Table 1:** The pseudo-first-order and pseudo-second-order linear kinetic models for As(III) sorption at 6 mg L^−1^ of initial concentration of As(III), sorbent dose 1 g L^−1^, and at 20 °C

Biochars	Pseudo-first-order model			Pseudo-second-order model		
	*q*_e_ (mg g^−1^)	*k*_1_(min^−1^)	*R*^2^	*q*_e_ (mg g^−1^)	*k*_2_ (g mg^−1^ min^−1^)	*R*^2^
Th/SCB–BC	3.13	0.06	0.34	2.89	0.34	0.99
ThRH–BC	3.10	0.06	0.36	2.73	0.36	0.99
SCB–BC	1.50	0.02	0.33	1.73	0.57	0.99
RH–BC	1.47	0.01	0.30	1.66	0.53	0.99

### Sorption isotherm modeling

All the non-linear sorption isotherm model parameters are shown in Table 2. The *R*^2^ value for the Freundlich model ranged from 0.88 to 0.96 for As(III) sorption on all four biochars (Fig. S3, Supplementary Information). The *Q*_F_ value of Th/SCB–BC was 1.2 mg^1−*n*^ g^−1^ L^*n*^ and it was 1.68, 1.80, and 2. mg^1−*n*^ g^−1^ L^*n*^ for Th/RH–BC, SCB–BC, and RH–BC, respectively. The sorption intensity (*1/n*) of Th/SCB–BC was relatively higher than the Th/RH–BC, SCB–BC, and RH–BC, indicating its higher sorption capacity for As(III).

**Table 2:** Non-linear sorption isotherm model parameters of Langmuir, Temkin, Dubinin–Radushkevich, and Freundlich models fit for As(III) sorption on untiolated and thiol-functionalized biochars at 6 mg L^−1^ initial concentration of As(III), sorbent doe 1 g L^−1^ and at 7 pH

Biochars	Langmuir			Freundlich			Temkin			Dubininin–Redushkevich		
	*Q*_L_ (mg g^−1^)	*K*_L_ (L g^−1^)	*R*^2^	*Q*_F_ (mg^1−n^ g^−1^ L^n^)	1/*n*	*R*^2^	*B*	*A*	*R*^2^	*Q*_D_ (mg g^−1^)	*E* (kJ g^−1^)	*R*^2^
Th/SCB–BC	1.49	7.93	0.99**	2.97	1.73	0.95**	0.75	48.0	0.89*	4.02	0.06	0.95**
Th/RH–BC	2.14	4.63	0.98**	0.50	1.30	0.96**	0.45	29.5	0.97**	1.79	0.06	0.96**
SCB–BC	1.19	5.63	0.96**	2.37	1.70	0.92**	0.88	18.7	0.91**	3.39	0.05	0.97**
RH–BC	0.88	3.19	0.93**	1.53	1.59	0.88*	0.77	10.5	0.94**	2.50	0.04	0.96**

*p < 0.05; **p < 0.0

In the case of Langmuir model, higher *R*^2^ (0.99) for Th/SCB–BC was observed than those of Th/RH–BC, SCB–BC, and RH–BC (0.98, 0.96, and 0.93, respectively) (Table 2; Fig. S4, Supplementary Information). Similarly, *Q*_L_ values for As(III) sorption on Th/SCB–BC and Th/RH–BC were also greater than the RH–BC and SCB–BC (Table 2). In this study, the Langmuir model was better than the Freundlich model in explaining As sorption, indicating that monolayer sorption was a dominant As sorption mechanism on the surface of all four sorbents (biochars) used in this study (Ali et al. 2020). Temkin model *R*^2^ values ranged from 0.89 to 0.97 for As(III) sorption by Th/SCB–BC, Th/RH–BC, SCB–BC, and RH–BC (Table 2; Fig. S5, Supplementary Information). Relatively lower heat of sorption (*b*) values were noted for As(III) sorption by Th/SCB–BC (0.75), Th/RH–BC (0.45), SCB–BC (0.88), and RH–BC (0.77), respectively, indicating that a linear decrease in *b* established a great coverage of As(III) on the surface of biochars (Foo and Hameed 2010).

The *R*^2^ values obtained in the Dubinin–Radushkevish isotherm model were between 0.95 and 0.97 on all four sorbents for As(III) (Table 2; Fig. S6, Supplementary Information). Bonding energy (*E*) was spanned 0.04 to 0.06 kJ g^−1^ (Table 2). It is well believed that if the *E* value is < 8 kJ g^−1^, then the sorption follows the physical process and pore-filling is a dominant mechanism. While if the *E* value is between 8–16 kJ g^−1^, chemisorption and ion exchange control the process (Niazi et al. 2018b). Based on our findings, the value of *E* ranges from 0.04 to 0.06 kJ g^−1^, suggesting that physical sorption may be the primary mechanism whereby As(III) rapidly occupies the available adsorption sites on the biochar surface. However, a lower *E* value represents that this model is not the best model to justify As(III) sorption on four biochar surfaces (Niazi et al. 2018a).

Isotherm results revealed that the Langmuir model with *R*^2^ = 0.99 was the best model to describe As(III) sorption on Th/SCB–BC where As(III) followed a monolayer sorption process indicating that the homogeneous distribution of functional groups on modified biochar surface play a major role in As(III) sorption.

### Desorption of As and sorbent reusability

**Fig. 3:**
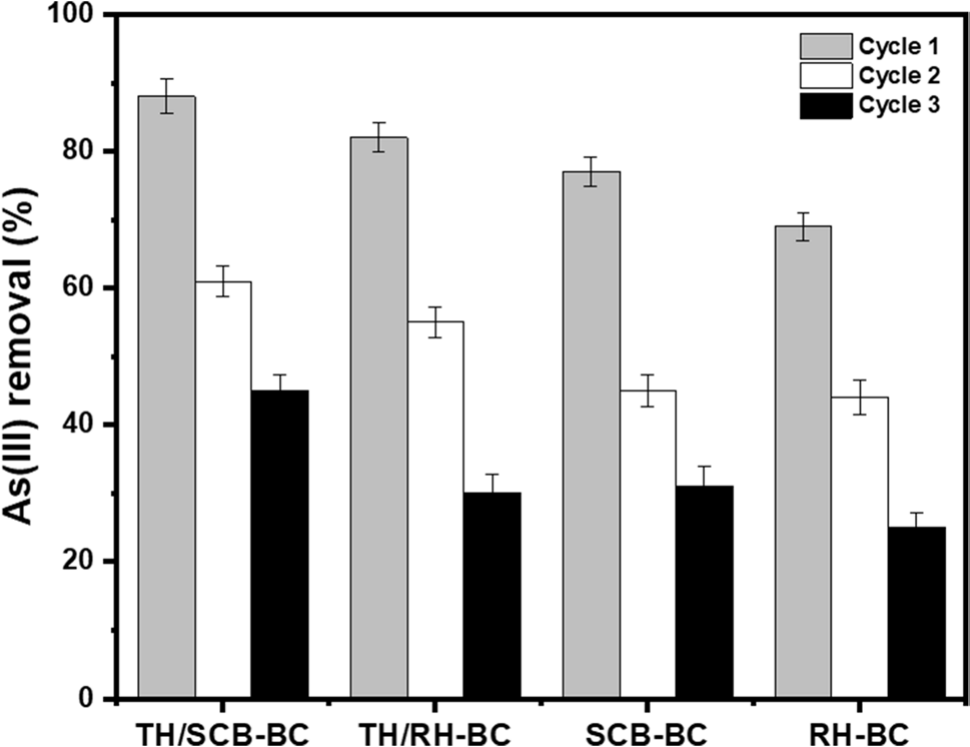
Desorption of As(III) from thiolated sugarcane bagasse (Th/SCB–BC), thiolated rice husk (Th/RH–BC), sugarcane bagasse biochar (SCB–BC), and rice husk biochar (RH–BC). Values are shown as mean ± standard error (*n* = 3)

An ideal sorbent should exhibit efficient post-use regeneration performance coupled with a cost-effective regeneration process (Moslehi et al. 2024). In this study, 0.2 M NaOH solution was applied to evaluate the stability and reusability of sorbents by desorbing As(III) in three cycles. Arsenic(III) removal efficiency of unthiolated and thiolated SCB and RH biochars decreased after the second and third cycles as follows: 61–45% on Th/SCB–BC, 55–30% on Th/RH–BC, 45–31% on SCB–BC, and 44–25% on RH–BC surface (Fig. 3).

Results showed that the percentage As(III) sorption capacity of prepared biochars decreased with sorption-desorption process up to three cycles. Higher percentage removal in the first cycle may be attributed to a higher number of available active sites. After the first sorption–desorption cycle, a rapid decline in As sorption was observed in the second and third regeneration cycles. This could possibly attributed to the saturation of sorbents (biochars) with -OH anions (negatively charged) from NaOH treatment resulting in competition with As oxyanions for sorption sites and decreasing As sorption ability of biochars over the second and third cycles (Iqbal et al. 2021; Xu et al. 2023).

### Sorbent characterization and possible mechanisms

#### Fourier-transform infrared (FTIR) spectroscopy

**Fig. 4:**
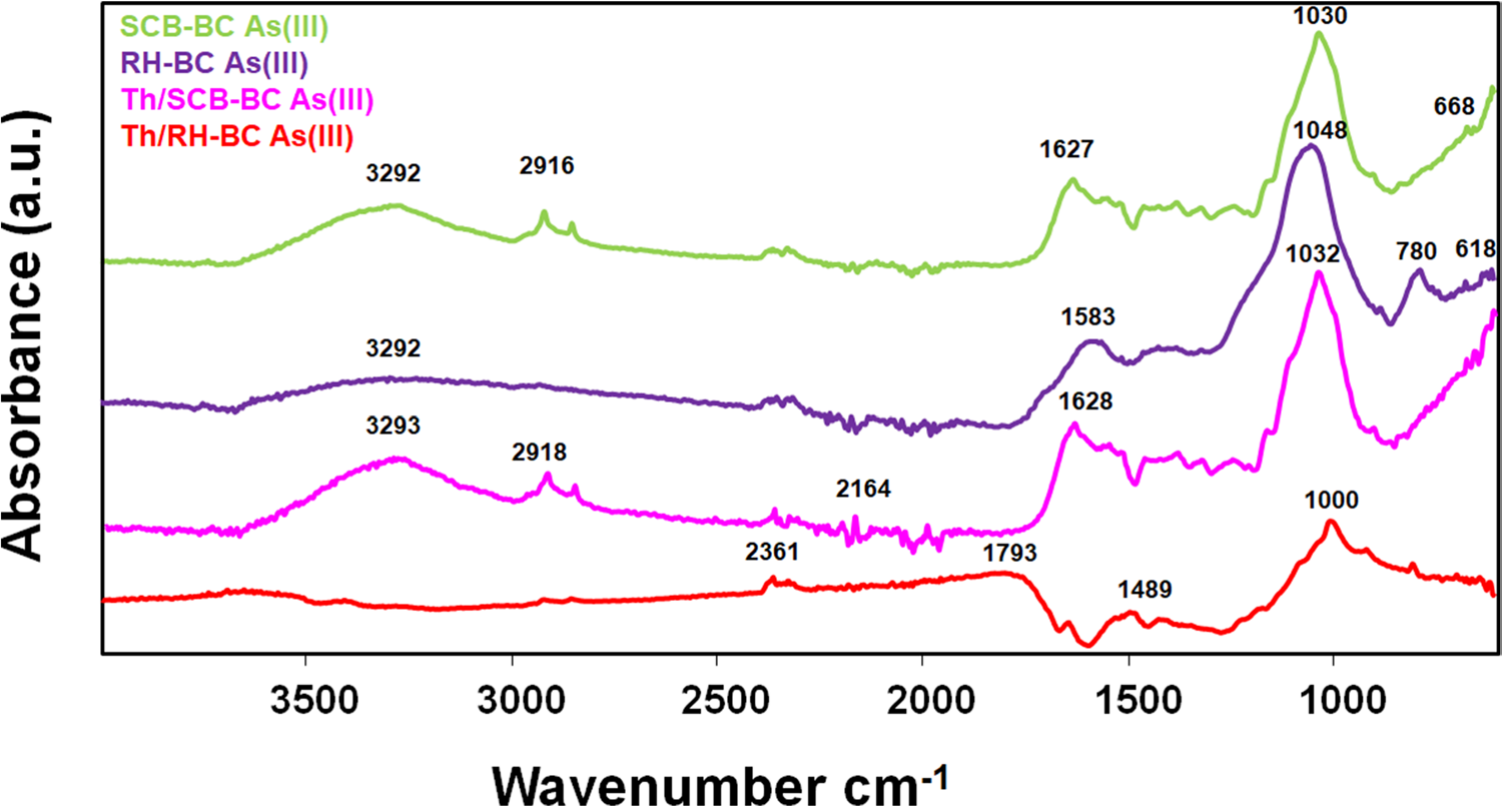
Fourier-transform infrared (FTIR) spectra of thiolated sugarcane bagasse biochar (Th/SCB–BC), Th/rice husk biochar (Th/RH–BC), sugarcane bagasse biochar (SCB–BC), and rice husk biochar (RH–BC) with As(III) loading at pH 7

The FTIR spectra of As(III)-loaded Th/SCB–BC, Th/RH–BC, SCB–BC, RH–BC, and As(III)-unloaded biochars are shown in Fig. 4 and Fig S7, Supplementary Information. In the case of As(III)-unloaded biochars, spectral peaks appeared at 3344 cm^−1^ (RH–BC) and 3269 cm^−1^ (SCB–BC) which corresponded to the stretching vibration of –OH groups (Niazi et al. 2018b). A small peak at 1793 cm^−1^ (SCB–BC) could be assigned to − C = O bonds and may be associated with carboxylic acids or their ester groups (Shakoor et al. 2018). Visible and sharp peaks appeared at 1029 cm^−1^ (SCB–BC) and 1043 cm^−1^ (RH–BC) indicating the − OH and − C–O stretching bands presence that showed a significant shift after As(III) loading (Huang et al. 2019) (Fig. 4; Fig S7, Supplementary Information).

In the case of As(III) loading (Fig. 4), spectral peaks at 3292 and 3293 cm^−1^ showed the presence of − OH groups, which indicated stretching vibration from − OH groups (Abu-Danso et al. 2017; Huang et al. 2019). The strong peak appeared at 1030 cm^−1^ and 1048 cm^−1^ for SCB–BC and RH–BC shifted to 1049 cm^−1^ (Th/SCB–BC) and 1008 cm^−1^ (Th/RH–BC), respectively, after the thiolation process. This may indicate the overlapping of − OH and − C–O stretching bands (Huang et al. 2019; Li Zhichao et al. 2013). The presence of peaks at 1489 and 1793 cm^−1^ on Th/RH–BC and 1628 cm^−1^ on Th/SCB–BC could be associated with − C = O vibration of the − COOH group and linked to carboxylic acid and or their ester groups, implying CH_4_N_2_S function containing − S–H groups (Wu et al. 2012). The spectral peaks at 1583 cm^−1^ correspond to − O–H bending for As(III)-loaded RH–BC sorption (Pazhoor et al. 2021). Small peaks at 2164 cm^−1^ (Th/SCB–BC) and 2361 cm^−1^ (Th/RH–BC) reflected the absorbance of − S–H groups, which demonstrated − S–H group grafting onto thiolated biochars (Li Zhichao et al. 2013; Lyu et al. 2020). The FTIR spectroscopy results suggested the grafting of − S–H functional groups, which may lead to higher As(III) sorption through direct surface complexation reaction on thiol-functionalized biochars.

#### X-ray photoelectron spectroscopy

**Fig. 5:**
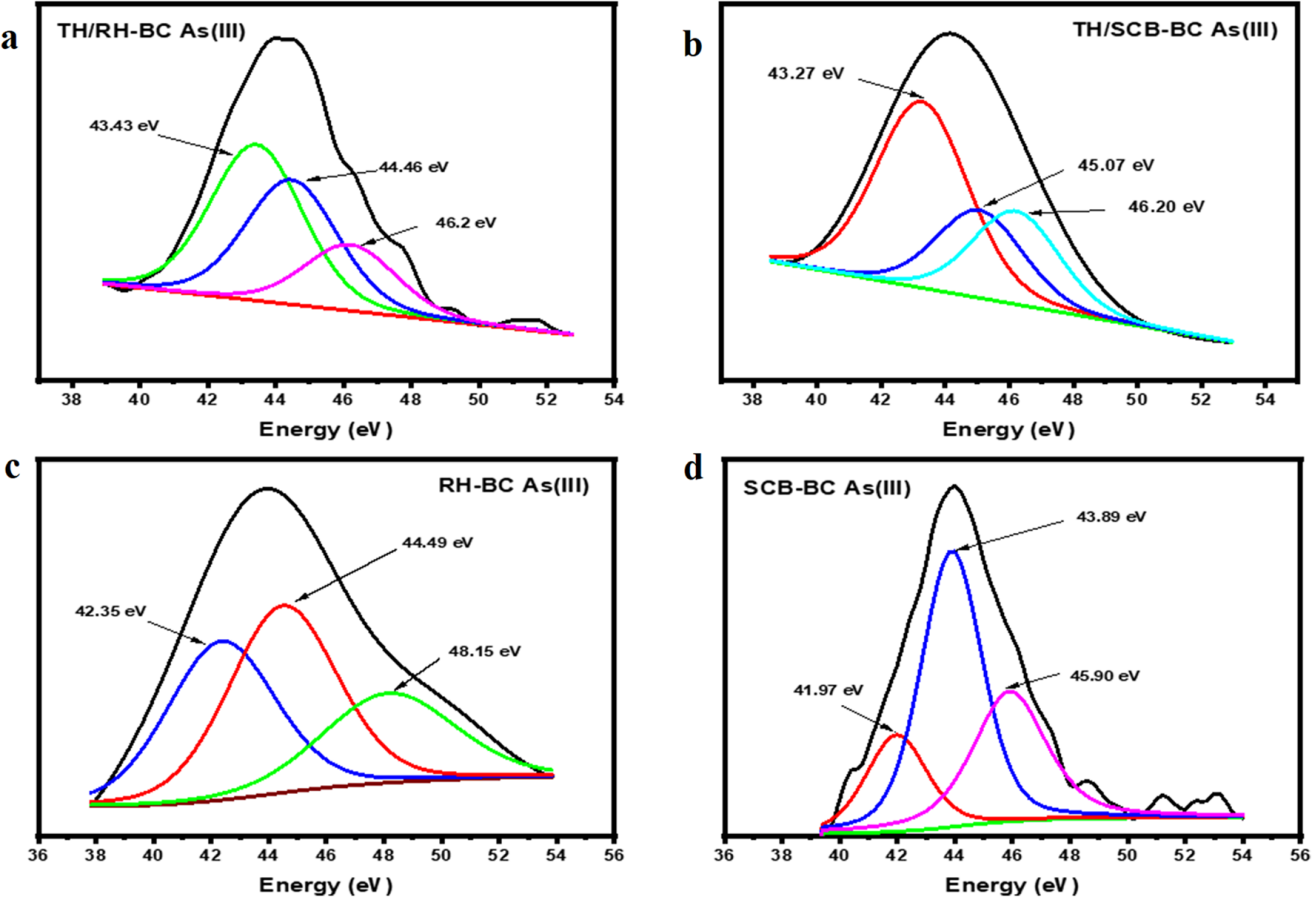
X-ray photoelectron spectroscopy (XPS) results obtained for As(III) sorption on (**a**) thiolated rice husk biochar (Th/RH–BC), **b** Th/sugarcane bagasse biochar (Th/SCB–BC), **c** rice husk biochar (RH–BC), and (**d**) sugarcane bagasse biochar (SCB–BC)

X-ray photon spectroscopy (XPS) was used for surface chemical analysis of sorbents and to identify As sorption with functional groups on biochar surfaces. The XPS spectra of biochars without As(III) and after As(III) sorption (Th/SCB–BC, Th/RH–BC, SCB–BC, RH–BC) are shown in Figs. 5 and S8 in the Supplementary Information. Results indicated the presence of surface elements such as C, N, O, and As(III) in pristine, Th/RH–BC, and As(III)-loaded RH (Fig. 5a, Fig. S8a, Supplementary Information). Typical peaks appeared at 284.7 eV (61.82%) and 286.17r eV (20.84%) for natural RH–BC, which showed a slight increase at 284.72 (61.99%) indicating the presence of C = C/C–C and decrease at 287.27 eV (8.93%) showing the presence of C = O/C–O, respectively, on Th/RH–BC controlling As sorption process.

There were two strong peaks at 529.96 eV (43.85%) and 531.29 (35.63%) for RH–BC, which showed a prominent change after As(III) sorption on Th/RH–BC at 529.99 eV (33.32%) and 531.4 eV (38.53%). This may indicate the role of O = C–O and carbonyl (O = C) groups. It has been reported that a peak at 42.2 eV indicates As(III) sorption (Sattar et al. 2019). The peak at 43.43 eV (45%) for Th/RH–BC As(III) and 44.49 (41.06%) for RH–BC As(III) corresponded to As(III) (Liu et al. 2022). The strength of peaks represents the presence of As(III) on biochars (Liu et al. 2022) (Fig. 5a, c). The binding energy of Th/RH–BC at 163.7 eV could be ascribed to the successful grafting of the − S–H group and depicted the presence of the As(III) − S–H bond (Fan et al. 2020). This peak could be attributed to chemisorbed dimethyl sulfide ((CH_3_)_2_S). Since the peak intensity is weaker after As(III) sorption, the methyl group might be replaced with As(III) (Zhang et al. 2015).

Figure S8b, Supplementary Information exhibited the XPS spectrum of As(III)-loaded Th/SCB–BC and SCB–BC, which indicated three characteristic peaks at 284 eV and 531 eV, possibly attributing to C 1 s and O 1 s. The C 1 s spectrum at 284.69 eV (46.15%) and 288 eV (37.14%) on SCB–BC showed an increase at 284.6 eV (76.10%) that is composed of C = C/C–C and a decrease at 286.7 eV (8.79%) corresponding to C = O/C–O groups, respectively, on Th/SCB–BC. The O 1 s spectrum showed two prominent peaks at 530.51 eV (46.20%) and 532.32 eV (45.77%) on the SCB–BC surface shifted to 530.32 eV (57.11%) and 531.46 eV (21.29%) after As(III) sorption on Th/SCB–BC (Pereira et al. 2014). This change in binding energy may represent the role of O = C–O and O = C groups. Similar to the Th/RH–BC, a weak peak appeared at 163.1 eV which could be assigned to the successful inclusion of the -S–H surface group and sorption of As(III) on Th/SCB–BC. Strong As(III) spectrum was shown at 43.27 (52.03%) and 43.89 eV (50.98%) on As(III)-loaded Th/SCB–BC and SCB–BC (Fig. 5b, d) showing the presence and As(III) on biochar surface. Overall, these findings are in agreement with the FTIR spectroscopy results, which indicated that there is a strong electrostatic interaction and surface complexes on thiolated biochars during As(III) sorption.

#### Scanning electron microscopy (SEM) and X-ray diffraction analysis (XRD)

The morphological features of the most promising biochars (i.e., unthiolated SCB-BC and Th/SCB–BC) were examined using SEM. Figure S9, Supplementary Information illustrates that the surface morphology was a rough and porous structure of biochar; notably, Th/SCB–BC surface micrographs showed a more porous structure with thiol-functionalization than other biochars (Fig. S9c and d, Supplementary Information). Figure S10 in Supplementary Information shows the XRD pattern of unthiolated and thiol-functionalized biochar, which showed the maximum As removal from water (Th/SCB–BC).

#### Possible mechanisms

The possible mechanisms of As(III) removal were proposed as (1) ligand exchange and surface complexation on biochar surface between surface functional groups (− S–H, − OH, − COOH, C = O) and As(III) anions; (2) chemical process may involve surface complexation with functional groups, such as − OH, carboxyl; and (3) electrostatic interaction between As(III) anions and mineral fraction containing calcium (Ca) in biochar as Ca–As precipitates (Niazi et al. 2018b).

## Conclusions

In this study, thiol-functionalized and unthiolated SCB and RH biochars were compared for the remediation of As-contaminated water. The FTIR spectroscopy indicated that surface functional groups, such as − OH, − COOH, and C = O were responsible for As(III) sorption onto biochars through surface complexation reactions. Notably, thiol-grafting-enhanced As(III) sorption by Th/SCB–BC and Th/RH–BC over their unthiolated biochars. The XPS analysis confirmed the presence of more As bonded with − S–H groups, especially on Th/SCB–BC and Th/RH–BC. The Th/SCB–BC showed higher sorption capacity whereas the Langmuir isotherm model (*R*^2^ = 0.99) and pseudo-second-order kinetic model (*R*^2^ = 0.99) were the best to fit experimental data. The findings of this study highlight that the Th/SCB–BC and Th/RH–BC could be a promising, cost-effective, and environmentally-friendly sorbents compared to SCB–BC and RH–BC for As(III) anions removal from aqueous solutions. Thus, − S–H modification increased the sorption capacity of As(III) onto Th/SCB–BC from As-contaminated water. Future research is warranted to evaluate the effect of varying biochar to thiourea ratio, co-exiting ions (e.g., sulfate, nitrate, chloride, phosphate), mixed As(III)–As(V) aquatic system on the As removal efficacy of thioated biochars, selective As removal and change in solution-phase As species. In future studies, it is intriguing to treat natural As-contaminated groundwater and perform a life cycle assessment analysis of the As treatment process in a real-world scenario prior to its large-scale application.

## Supplementary Information

Below is the link to the electronic supplementary material.

Supplementary file1 (DOCX 2166 KB)

## Data Availability

All additional data are presented in the Supporting Information. Other data attached to the paper, if any, may be available upon reasonable request.
