# Supplementary material for: Enhanced capacity of thiol-functionalized sugarcane bagasse and rice husk biochars for arsenite sorption in aqueous solutions
Source: Environ Sci Pollut Res Int. 2024 Aug 15;31(39):52293–305. doi: 10.1007/s11356-024-34661-4 (PMC11374841; doi:10.1007/s11356-024-34661-4)
Supplement: Supplementary file 1 — Supplementary file1 (DOCX 2166 KB) [file 11356_2024_34661_MOESM1_ESM.docx]

**SUPPLEMENTARY INFORMATION**

### Enhanced capacity of thiol-functionalized sugarcane bagasse and rice husk biochars for arsenite sorption in aqueous solutions

Israr Masood ul Hasan^1,2,3^, Nabeel Khan Niazi^1^, Irshad Bibi^4,5,^*, Fazila Younas^6^, Fahad Al–Misned^7^, Muhammad Bilal Shakoor^8^, Fawad Ali^9,10^, Shazia Ilyas^11^, Muhammad Mahroz Hussain^1^, Jinli Qiao^2,12^, Andreas Lüttge^4^

*^1^ Institute of Soil and Environmental Sciences, University of Agriculture Faisalabad, Faisalabad, 38040, Pakistan (E–mail:* [*israrmasood53@yahoo.com*](mailto:israrmasood53@yahoo.com)*; nabeelkniazi@gmail.com; hmahroz@gmail.com)*

*^2^ State Key Laboratory for Modification of Chemical Fibers and Polymer Materials, College of Environmental Science and Engineering, Donghua University, 2999 Ren’min North Road, Shanghai 201620, China (E–mail:* [*qiaojl@dhu.edu.cn*](mailto:qiaojl@dhu.edu.cn)*)*

*^3^Key Laboratory of Comprehensive and Highly Utilization of Salt and Lake Resources, Qinghai Institute of Salt and Lakes, Chinese Academy of Sciences, Xining, 810008, China*

*^4^ Department of Geosciences and MARUM—Center for Marine Environmental Sciences, University of Bremen, Bremen 28359, Germany (irshad.niazi81@gmail.com; aluttge@marum.de)*

*^5^ School of Geography, Earth and Atmospheric Sciences, University of Melbourne, Melbourne, VIC, 3053, Australia*

*^6^ School of Environmental Science and Engineering, Shandong University, Qingdao, 266237, China (*[*fazila.younas@gmail.com*](mailto:fazila.younas@gmail.com)*)*

*^7^ Department of Zoology, College of Science, King Saud University, Riyadh 11451, Saudi Arabia (*[*almisned@ksu.edu.sa*](mailto:almisned@ksu.edu.sa)*)*

*^8^ College of Earth and Environmental Sciences, University of the Punjab, Lahore 54000, Pakistan (E–mail:* [*bilalshakoor88@gmail.com(BS)*](about:blank)*)*

*^9^ Centre for Planetary Health and Food Security, Griffith University, Nathan campus (4111), Brisbane, Queensland–Australia (E–mail: fawadniazi_a@yahoo.com(FA))*

*^10^ Queensland Department of Agriculture and Fisheries (QDAF), Mareeba (4880), Queensland–Australia*

*^11^ Department of Environmental Sciences, Forman Christian College (A Chartered University), 54600 Lahore, Pakistan (shaziailyas@fccollege.edu.pk)*

*^12^ Shanghai Institute of Pollution Control and Ecological Security, Shanghai 200092, China*

*Corresponding authors’ footnote:*

*Irshad Bibi, School of Geography, Earth and Atmospheric Sciences, University of Melbourne, Melbourne, VIC, 3053, Australia; Email: irshad.niazi81@gmail.com; irshad.bibi@unimelb.edu.au

**Table S1:** Comparison of maximum sorption of As(III) onto different sorbents in batch sorption experiments with thiolated sugarcane bagasse (Th/SCB-BC), thiolated rice husk (Th/RH-BC), sugarcane bagasse biochar (SCB-BC) and rice husk biochar (RH-BC) used in this study.

| **Sorbents** | **Sorbent dose (g/L)** | **Initial concentration (mg/L)** | **Optimum pH** | **Maximum sorption**  **(mg g^-1^)** | **References** |
| --- | --- | --- | --- | --- | --- |
| Perilla leaf biochar | 1 | 0.05 to 7.0 | 8 | 3.20 (As(III) | (Niazi *et al.*, 2018a) |
| Japanese oak wood biochar | 1 | 0.05 to 7.0 | 7.3 | 1.81 (As(III)) | (Niazi *et al.*, 2018b) |
| Water melon rind | 1 | 4 | 8.2 | 3.40 (As(III)) | (Shakoor *et al.*, 2018) |
| Almond shell | 0.6 | 0.01 to 7 | 7.2 | 4.6 (As(III)) | (Ali *et al.*, 2020) |
| Xanthated water melon rind | 1 | 5 | 4.6 | 3.96 (As(V)) | (Shakoor *et al.*, 2018) |
| Almond shell biochar | 0.6 | 0.01 to 7 | 7.2 | 4.86 (As(III)) | (Ali *et al.*, 2020) |
| Thioglycolated sugarcane carbon | 0.05 | 50–500 μg/L | 7 | 0.085 (As(III)) | (Roy *et al.*, 2014) |
| Clay pellets | 10 | 0.1 to 100 | 7 | 0.4 (As(V)) | (Thanh *et al.*, 2019) |
| Cawfish shell biochar | 0.1 | 20 to 80 | 1.48 | 17.2 (As(V)) | (Yan *et al.*, 2018) |
| Chitosan | 5 | 1 | 4.5 | 19.6 (As(V)) | (Mora *et al.*, 2019) |
| Charred orange peel | 4 | 200 | 6.5 | 60.9 (As(V)) | (Abid *et al.*, 2016) |
| Pine cone (PC) biochar | 10 | 50 to 200 μg/L | 4 | 0.0070 (As(III)) | (Van Vinh et al. 2015) |
| Zinc-loaded PC biochar | 10 | 50 to 200 μg/L | 4 | 0.0057 (As(III)) | (Van Vinh et al. 2015) |
| Thiolated Sugarcane bagasse biochar | 1 | 6 | 7 | 2.75 (As(III)) | This study |
| Thiolated Rice husk biochar | 1 | 6 | 7 | 2.43 (As(III)) | This study |
| Sugarcane bagasse biochar | 1 | 6 | 6 | 1.94 (As(III)) | This study |
| Rice husk biochar | 1 | 6 | 6 | 1.65 (As(III)) | This study |


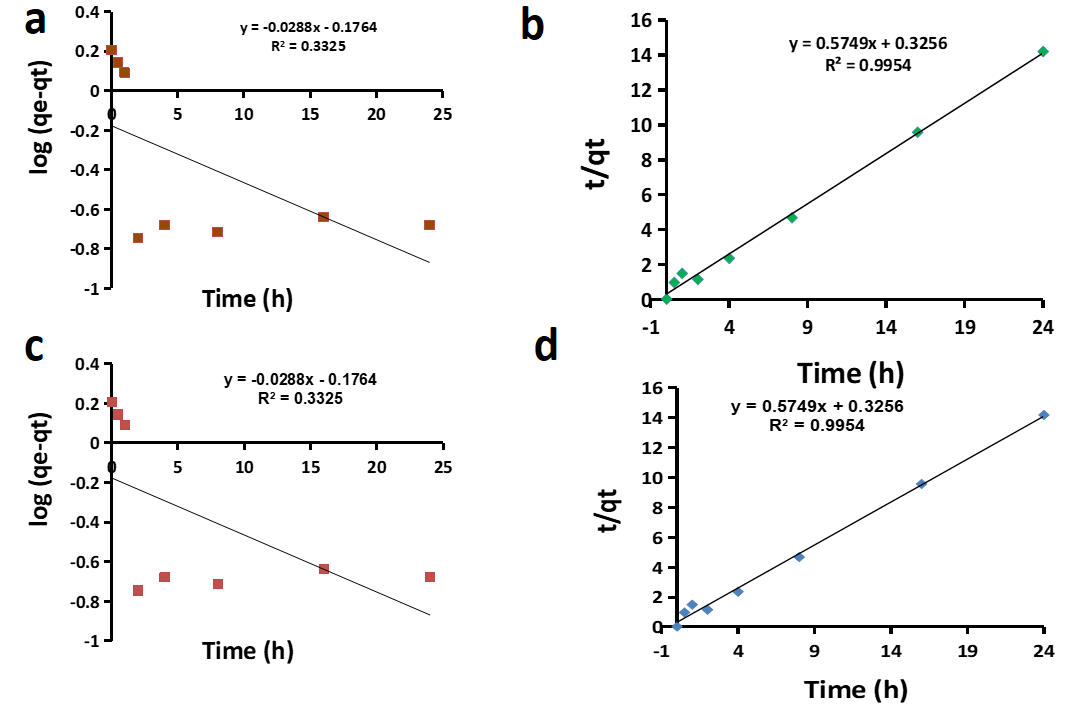


**Fig. S1** The linearized plots of pseudo-first order model for As(III) (a) and pseudo-second order model for As(III) sorption (b) of RH-BC and pseudo-first order model for As(III) (c) and pseudo-second order model for As(III) sorption (d) of SCB-BC.


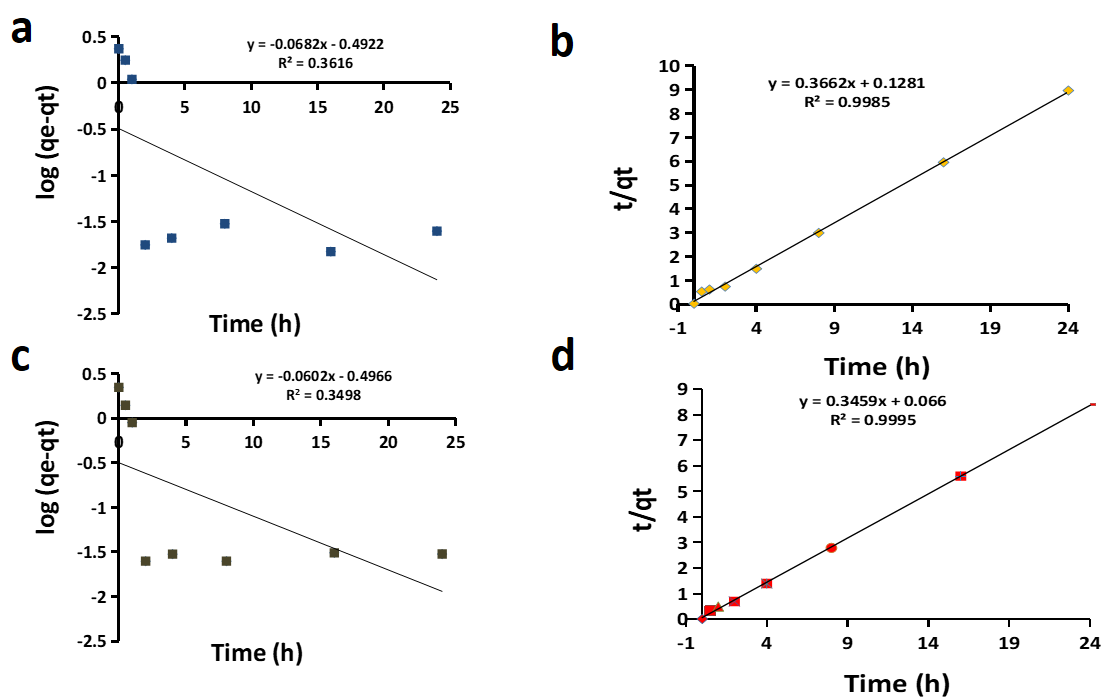


**Fig. S2** The linearized plots of pseudo-first order model for As(III) (a) and pseudo-second order model for As(III) sorption (b) of Th/RH-BC and pseudo-first order model for As(III) (c) and pseudo-second order model for As(III) sorption (d) of Th/SCB-BC.


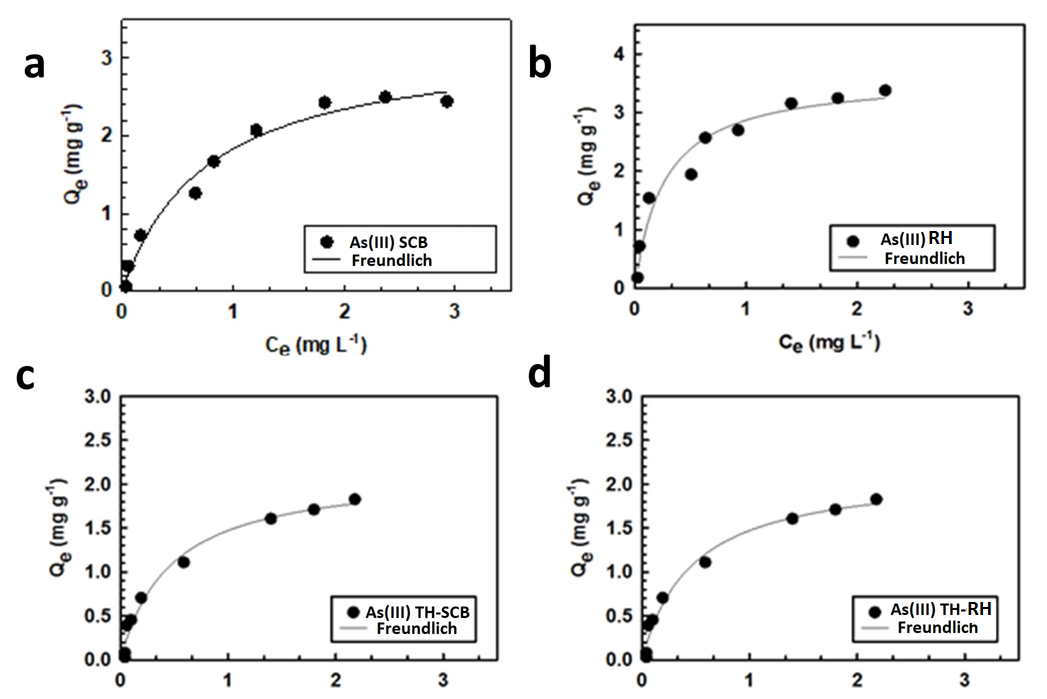


**Fig. S3** Freundlich sorption isotherms of thiolated sugarcane bagasse (Th/SCB-BC), thiolated rice husk (Th/RH-BC), sugarcane bagasse biochar (SCB-BC) and rice husk biochar (RH-BC) As(III) at pH 4-7, sorbent dose = 1 g/L, and *T* = 20 ^o^C. The solid (−) line represents the model fits of the experimental data and black circles (●) indicate experimental data.


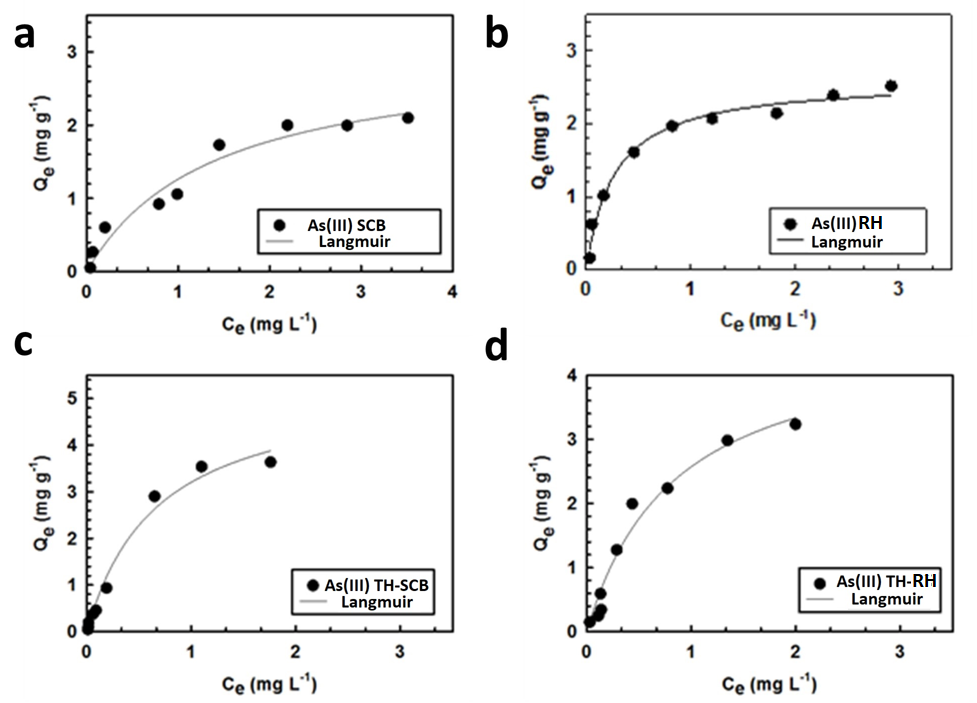


**Fig. S4** Langmuir sorption isotherms of thiolated sugarcane bagasse (Th/SCB-BC), thiolated rice husk (Th/RH-BC), sugarcane bagasse biochar (SCB-BC) and rice husk biochar (RH-BC)As(III) at pH 4-7, sorbent dose = 1 g/L, and *T* = 20 ^o^C. The solid (−) line represents the model fits of the experimental data and black circles (●) indicate experimental data.


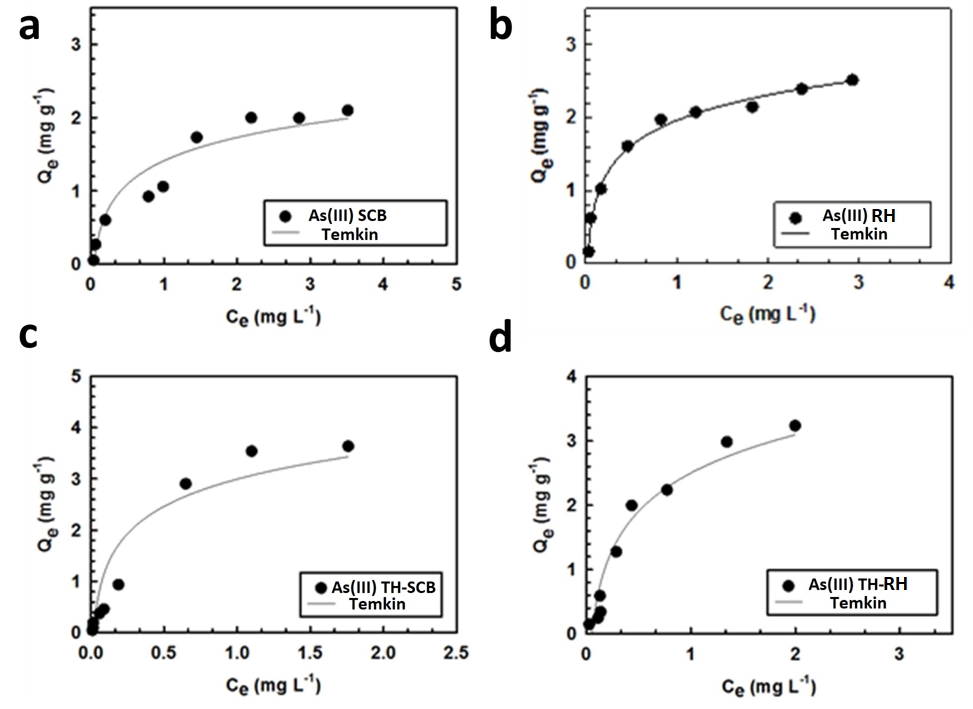


**Fig. S5** Temkin sorption isotherms of thiolated sugarcane bagasse (Th/SCB-BC), thiolated rice husk (Th/RH-BC), sugarcane bagasse biochar (SCB-BC) and rice husk biochar (RH-BC)As(III) at pH 4-7, sorbent dose = 1 g/L, and *T* = 20 ^o^C. The solid (−) line represents the model fits of the experimental data and black circles (●) indicate experimental data


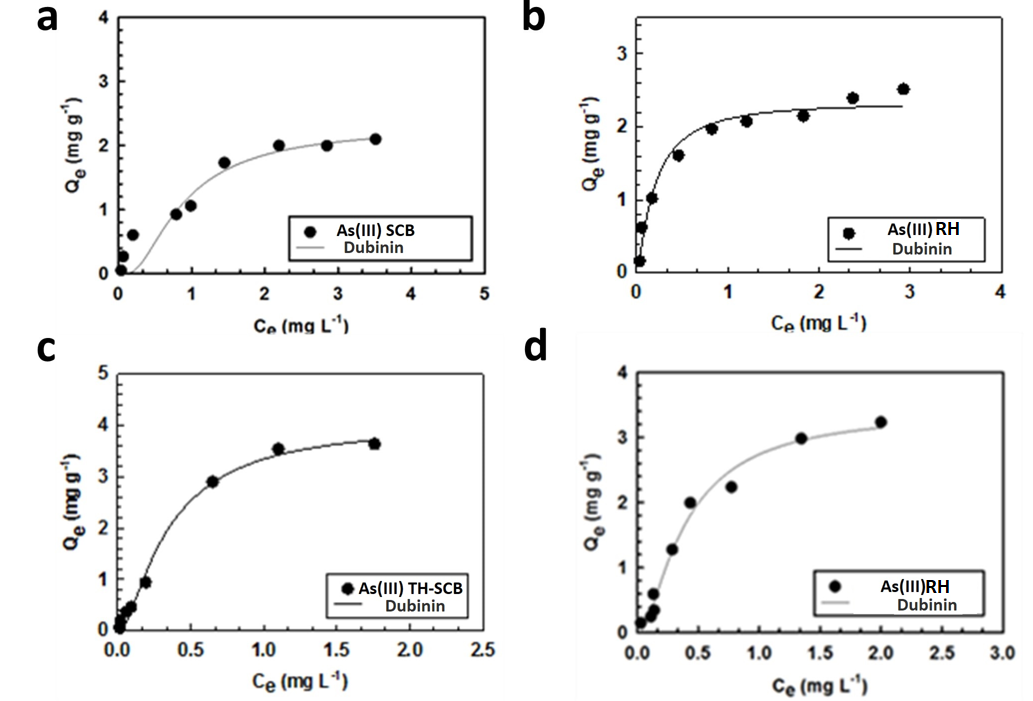


**Fig. S6** Dubinin-Radushkevich isotherms of thiolated sugarcane bagasse (Th/SCB-BC), thiolated rice husk (Th/RH-BC), sugarcane bagasse biochar (SCB-BC) and rice husk biochar (RH-BC) As(III) at pH 4-7, sorbent dose = 1 g/L, and *T* = 20 ^o^C. The solid (−) line represents the model fits of the experimental data and black circles (●) indicate experimental data


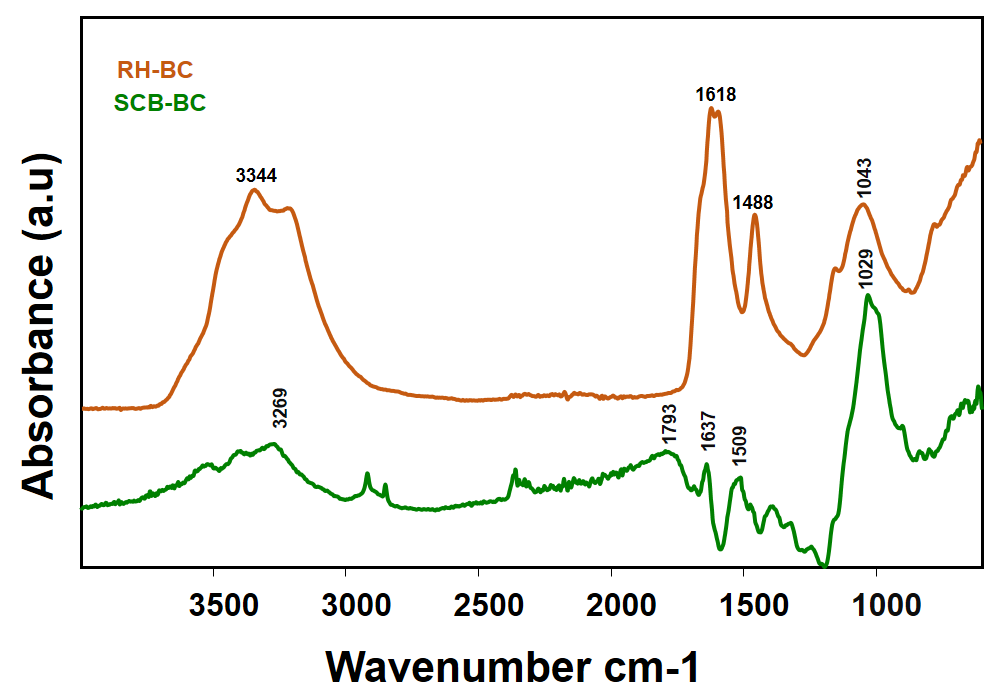


**Fig. S7** Fourier transform infrared (FTIR) absorbance spectra of sugarcane bagasse biochar (SCB-BC) and rice husk biochar (RH–BC) without As(III)–loading at pH 7.


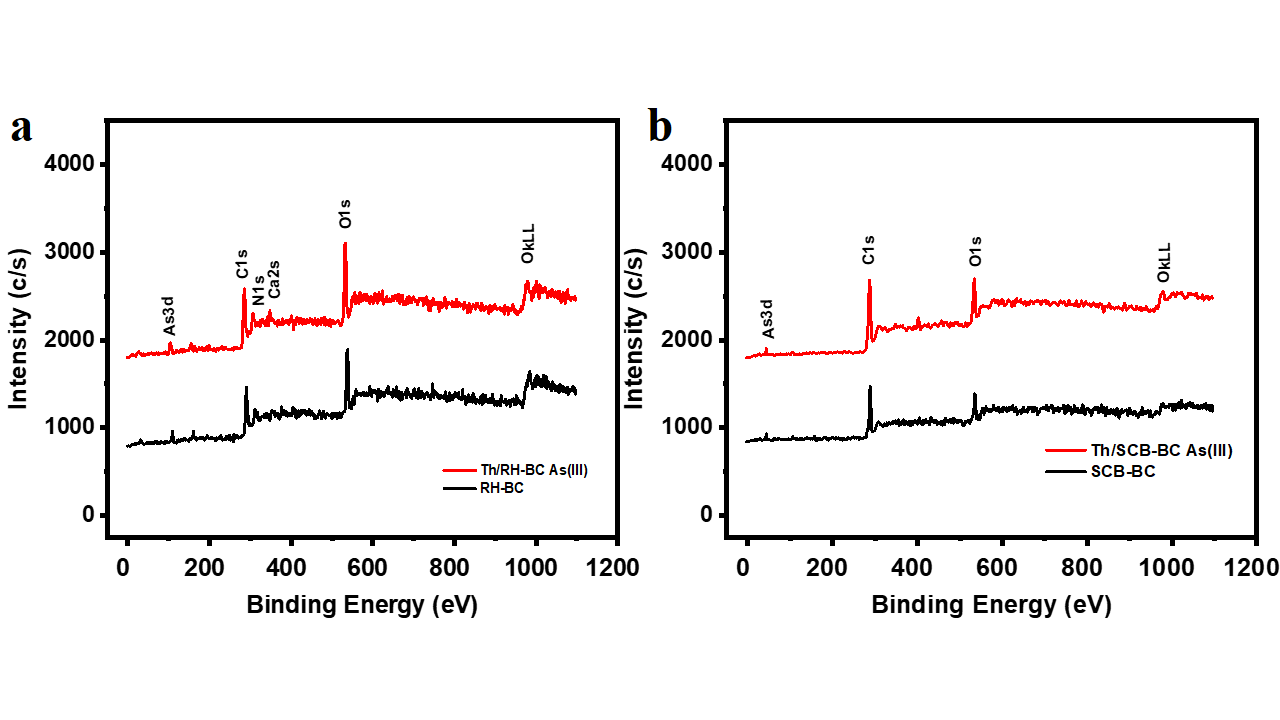


**Fig. S8** X-ray photoelectron spectroscopy (XPS) **a)** Th/RH-BC, RH-BC b) Th/SCB-BC, SCB-BC at pH 7.


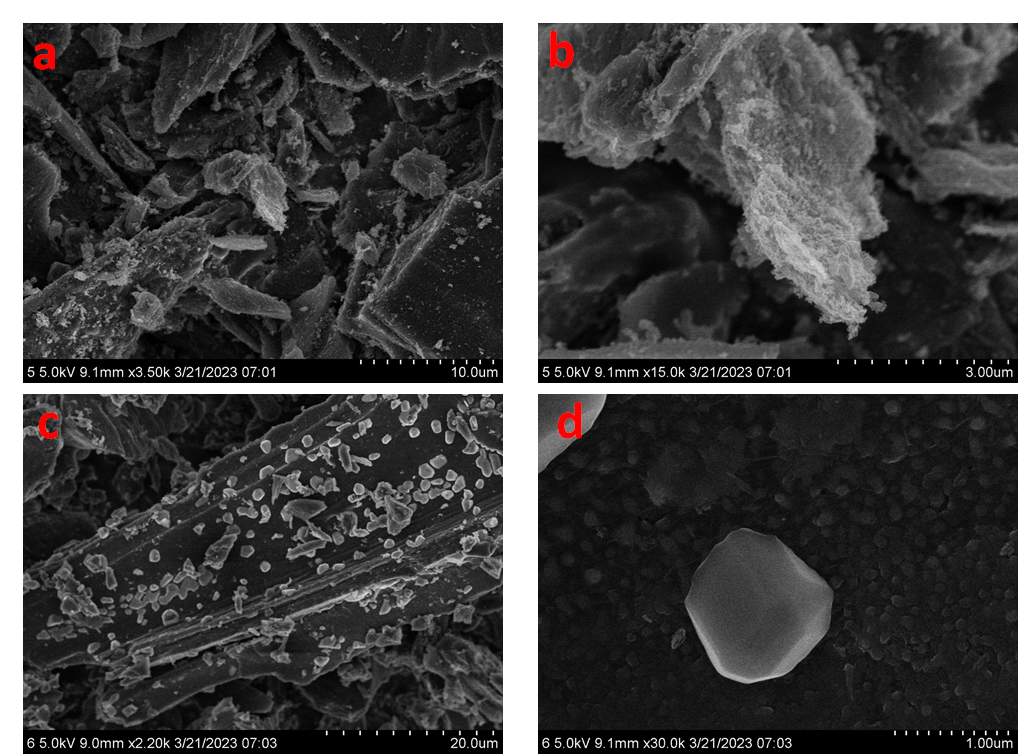


**Fig. S9** Scanning electron microscopy (SEM) images of SCB-BC (a,b) and Th/SCB-BC (c,d ) at pH 7.


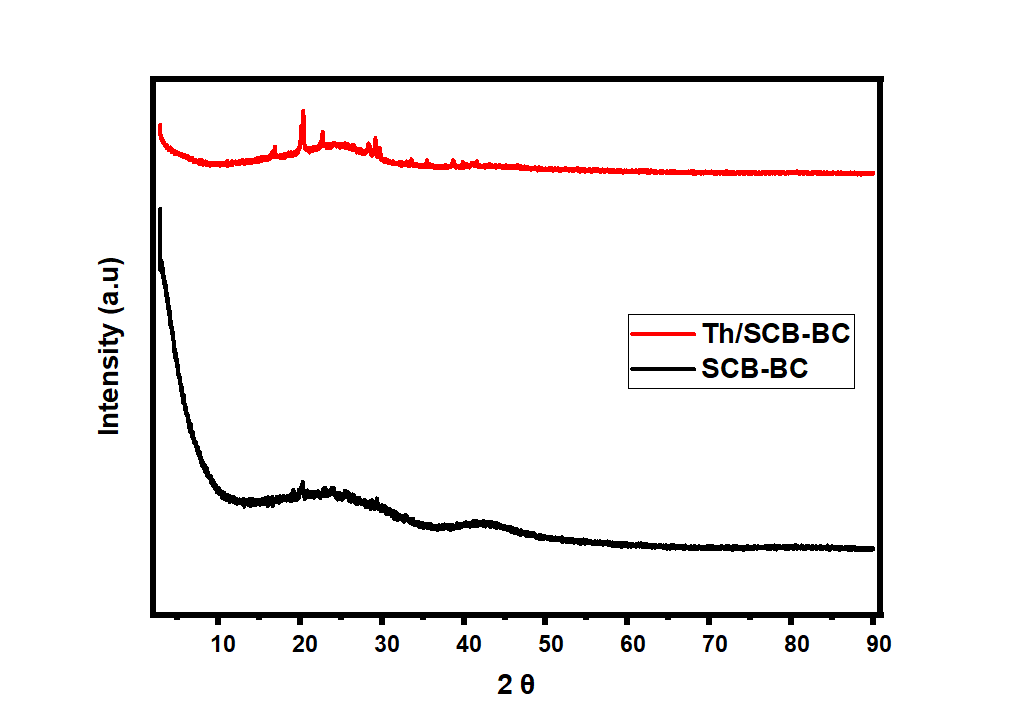


**Fig. S10** X-ray Diffraction (XRD) of SCB-BC and Th/SCB-BC at pH 7

**Details of sorption isotherm models**

Two parameter-based equilibrium isotherm models of Freundlich, Langmuir, Temkin, and Dubinin–Radushkevich were applied to describe the sorption mechanism of As(III) on the surface of prepared biochars. Freundlich model describes sorption on heterogeneous surfaces (multi-layer sorption) and can be expressed as (Eq. 1) (Ahmad et al. 2013):

$$Q_{e}= Q_{F}C_{e}^{1/n} (1)$$

where *Q_F_* is the relative As sorption capacity (mg g^-1^), and 1/*n* is a parameter related to sorption intensity or linearity.

The sorption on planar surfaces and monolayer sorption are described by the Langmuir model (Eq. 2):

$$Q_{e}= Q_{L}K_{L}C_{e}(1+K_{L}C_{e})^{-1}(2)$$

where *Q_L_* is the maximum amount of As sorbed (mg g^-1^), and *K_L_* is the equilibrium constant (L mg^-1^). Temkin model is related to the heat of sorption (Eq. 3):

$$Q_{e}=RT/b\ln\left( AC_{e} \right) (3)$$

where *R* is the universal gas constant, *T* is the absolute temperature, *b* is the heat of sorption, and *A* is the binding constant (L mg ^-1^). The heat of sorption, *b*, is an important parameter to differentiate between the sorption efficiency of sorbents (Ashraf et al. 2017).
The Dubinin–Radushkevich model describing sorption onto porous structure of the sorbent is represented as follows (Eq. 4) (Ahmad et al. 2013):

$Q_{e}= Q_{D}exp(- B_{D}[RT\ln$(1 + 1/$C_{e}$)$]^{2})$ (4)

where *Q_D_* is the adsorption capacity (mg g^-1^), *B_D_* is the mean free energy of sorption, and *E* is the bonding energy for the ion-exchange mechanism calculated using Eq. 5:

$$E=1/(2B_{D})^{0.5} (5)$$

The essential features of the Langmuir model are expressed in terms of a separation factor (*R_L_*) to determine whether the sorption was favorable or unfavorable in batch experiments, and is given by Eq. 6:

$$R_{L}=1/(1+ K_{L}C_{o}) (6)$$

**References**

Niazi, N.K., I. Bibi, M. Shahid, Y.S. Ok, E.D. Burton, H. Wang, S.M. Shaheen, J. Rinklebe and A. Lüttge. 2018a. Arsenic removal by perilla leaf biochar in aqueous solutions and groundwater: An integrated spectroscopic and microscopic examination. Environ. Pollut. 232:31-41.

Niazi, N.K., I. Bibi, M. Shahid, Y.S. Ok, S.M. Shaheen, J. Rinklebe, H. Wang, B. Murtaza, E. Islam and M.F. Nawaz. 2018b. Arsenic removal by Japanese oak wood biochar in aqueous solutions and well water: Investigating arsenic fate using integrated spectroscopic and microscopic techniques. Sci. Total Environ. 621:1642-1651.

Shakoor, M.B., N.K. Niazi, I. Bibi, M. Shahid, F. Sharif, S. Bashir, S.M. Shaheen, H. Wang, D.C. Tsang and Y.S. Ok. 2018. Arsenic removal by natural and chemically modified water melon rind in aqueous solutions and groundwater. Sci. Total Environ. 645:1444-1455

Ali, S., M. Rizwan, M.B. Shakoor, A. Jilani and R. Anjum. 2020. High sorption efficiency for as (iii) and as (v) from aqueous solutions using novel almond shell biochar. Chemosphere. 243:125330.

Roy, P., N. Mondal and K. Das. 2014. Modeling of the adsorptive removal of arsenic: A statistical approach. J. Environ. Chem. Eng. 2:585-597.

Thanh, N.C., B. Wichitsathian, C. Yossapol, W. Wonglertarak and B. Te. 2019. Improvement of aqueous solution coexisting with arsenite and arsenate using iron mixed porous clay pellets in batch and fixed-bed column studies. Water Supply. 19:1929-1937.

Yan, J., Y. Xue, L. Long, Y. Zeng and X. Hu. 2018. Adsorptive removal of as (v) by crawfish shell biochar: Batch and column tests. Environ. Sci. Pollut. Res. 25:34674-34683.

Mora, B.P., S. Bellú, M.F. Mangiameli, M.I. Frascaroli and J.C. González. 2019. Response surface methodology and optimization of arsenic continuous sorption process from contaminated water using chitosan. J. Water Process Eng. 32:100913.

Abid, M., N.K. Niazi, I. Bibi, A. Farooqi, Y.S. Ok, A. Kunhikrishnan, F. Ali, S. Ali, A.D. Igalavithana and M. Arshad. 2016. Arsenic (v) biosorption by charred orange peel in aqueous environments. Int. J. Phytorem. 18:442-449.

Ahmad M, Lee SS, Rajapaksha AU, Vithanage M, Zhang M, Cho JS, Lee S-E, Ok YS (2013): Trichloroethylene adsorption by pine needle biochars produced at various pyrolysis temperatures. Bioresour. Technol. 143, 615-622

Ashraf A, Bibi I, Niazi NK, Ok YS, Murtaza G, Shahid M, Kunhikrishnan A, Li D, Mahmood T (2017): Chromium(VI) sorption efficiency of acid-activated banana peel over organo-montmorillonite in aqueous solutions. Int. J. Phytorem., <http://dx.doi.org/10.1080/15226514.2016.1256372>

Van Vinh N, Zafar M, Behera SK, Park HS (2015): Arsenic(III) removal from aqueous solution by raw and zinc-loaded pine cone biochar: equilibrium, kinetics, and thermodynamics studies. International Journal of Environmental Science and Technology 12, 1283-1294
